# EEG alpha activity reflects motor preparation rather than the mode of action selection

**DOI:** 10.3389/fnint.2012.00059

**Published:** 2012-08-14

**Authors:** Marie-Pierre Deiber, Etienne Sallard, Catherine Ludwig, Catherine Ghezzi, Jérôme Barral, Vicente Ibañez

**Affiliations:** ^1^INSERM U1039, Faculty of MedicineLa Tronche, France; ^2^Clinical Neurophysiology and Neuroimaging Unit, Department of Psychiatry, University Hospitals of GenevaChêne-Bourg, Geneva, Switzerland; ^3^GRISSUL, University of LausanneLausanne, Switzerland; ^4^FPSE, University of GenevaGeneva, Switzerland; ^5^CIG, University of GenevaGeneva, Switzerland

**Keywords:** motor preparation, motor selection, externally-cued action, internally-cued action, alpha-band activity, motor-related amplitude asymmetry

## Abstract

Alpha-band activity (8–13 Hz) is not only suppressed by sensory stimulation and movements, but also modulated by attention, working memory and mental tasks, and could be sensitive to higher motor control functions. The aim of the present study was to examine alpha oscillatory activity during the preparation of simple left or right finger movements, contrasting the external and internal mode of action selection. Three preparation conditions were examined using a precueing paradigm with S1 as the preparatory and S2 as the imperative cue: Full, laterality instructed by S1; Free, laterality freely selected and None, laterality instructed by S2. Time-frequency (TF) analysis was performed in the alpha frequency range during the S1–S2 interval, and alpha motor-related amplitude asymmetries (MRAA) were also calculated. The significant MRAA during the Full and Free conditions indicated effective external and internal motor response preparation. In the absence of specific motor preparation (None), a posterior alpha event-related desynchronization (ERD) dominated, reflecting the main engagement of attentional resources. In Full and Free motor preparation, posterior alpha ERD was accompanied by a midparietal alpha event-related synchronization (ERS), suggesting a concomitant inhibition of task-irrelevant visual activity. In both Full and Free motor preparation, analysis of alpha power according to MRAA amplitude revealed two types of functional activation patterns: (1) a motor alpha pattern, with predominantly midparietal alpha ERS and large MRAA corresponding to lateralized motor activation/visual inhibition and (2) an attentional alpha pattern, with dominating right posterior alpha ERD and small MRAA reflecting visuospatial attention. The present results suggest that alpha oscillatory patterns do not resolve the selection mode of action, but rather distinguish separate functional strategies of motor preparation.

## Introduction

Selection and preparation of motor action constitute a subset of executive functions necessary for goal-directed behavior, and have received considerable interest in neuroscience research. From monkey electrophysiological data to brain imaging studies, the neuro-anatomical network involved in motor selection and preparation has been extensively examined, highlighting the key role of supplementary motor area (SMA), pre-SMA, anterior cingulate, dorsolateral prefrontal, dorsal premotor and posterior parietal cortex (Hoffstaedter et al., 2012 for review). The motor priming paradigm (Rosenbaum and Kornblum, 1982), which uses a preparatory stimulus (S1) conveying information about an upcoming movement, later cued by an imperative stimulus (S2), has been widely employed to disentangle the phases of preparation and execution of movement. In the interval between S1 and S2, averaging of the surface S1-locked EEG activity elicits a slow negative potential (contingent negative variation, Tecce and Cattanach, 1991), which amplitude is proportional to the number of movement dimensions specified in S1 (Bonnet and MacKay, 1989; Ulrich et al., 1998; Leuthold et al., 2004; Deiber et al., 2005). In complement to event-related potentials, frequency-specific EEG oscillatory reactivity provides a valuable tool for evaluating the temporal dynamics of activation in neuronal networks. Brain oscillations are believed to subtend the transfer of information across cerebral regions and support the binding mechanisms involved in sensory, motor and cognitive processing (Singer, 1993; Engel et al., 2001; Salinas and Sejnowski, 2001). In sensorimotor functions, modulations of EEG activity have been predominantly described in the alpha (8–13 Hz) and beta (14–30 Hz) frequency ranges (Pfurtscheller, 1981; Toro et al., 1994). A decrease in alpha or beta oscillatory activity relative to a baseline level indicates a state of active cortical processing and is generally referred to as an event-related desynchronization (ERD), as opposed to an increase, which is described as an event-related synchronization (ERS) (Pfurtscheller and Andrew, 1999).

Studies of oscillatory activities in S1–S2 delayed response tasks have consistently reported an alpha and beta ERD over the sensorimotor regions after S1, the amplitude of which depended primarily on whether or not a response was required at S2 (Go as opposed to No Go), as well as on S1 informative content (Kaiser et al., 2001; Alegre et al., 2004; Deiber et al., 2005; Klostermann et al., 2007; Tzagarakis et al., 2010; Sabate et al., 2012). Lateralized alpha and beta ERD is observed before and during execution of unilateral movements, reflecting the lateralization of spectral power decrease over the sensorimotor regions contralateral to the responding hand (Pfurtscheller, 1981; Arroyo et al., 1993; Pfurtscheller and Andrew, 1999). Such lateralization can be evaluated by computation of the motor-related amplitude asymmetry (MRAA), or lateralized ERD index, which measures the difference between contra- and ipsilateral oscillatory activities in specified frequency bands. In the S1–S2 preparation interval, analysis of this asymmetry index has revealed significant lateralization of alpha and beta ERD over sensorimotor regions when reliable information was provided regarding the upcoming response hand (Doyle et al., 2005; de Jong et al., 2006; Gladwin et al., 2008; Yamanaka and Yamamoto, 2010).

Two modes of motor selection have been commonly distinguished over the past 20 years: externally-driven actions, in response to external specification, and internally-driven or self-generated actions, based on self-decision (free choice) about the action (Passingham, 1987). Accumulating evidence from electrophysiological (Okano and Tanji, 1987; Romo and Schultz, 1987; Mushiake et al., 1991), neuroimaging (Deiber et al., 1991, 1996, 1999; Jahanshahi et al., 1995; Cunnington et al., 2002), and EEG studies (Papa et al., 1991; Jahanshahi et al., 1995; Gerloff et al., 1998; Thut et al., 2000) suggests partially distinct cerebral processes associated with each mode of action selection. Externally-cued actions preferentially engage the lateral premotor system (dorsal premotor cortex and cerebellum), while internally-driven actions may be principally mediated by the medial frontal system (SMA and basal ganglia). Regarding brain oscillations, little is known about the response of neural populations in each mode of action selection. To our knowledge, only one study has addressed the question using a simple Reaction Time (RT) design in a small population, showing differential reactivity of alpha and beta frequency bands to sub-processes of attention as opposed to selection and execution (Tremblay et al., 2008). However, the use of simple RT tasks requiring immediate response to instruction limits adequate analysis of motor selection and preparation. Moreover, as a random choice among four possible responses, the internal task used in the aforementioned study was probably more complex than the externally-cued task, which consisted of a single stimulus-response association. Indeed, a current debate concerns the issue of higher demands on working memory, conflict processing and task complexity inherent to self-initiated as compared to externally-cued actions, potentially limiting the validity of comparative interpretation (Nachev et al., 2008; Passingham et al., 2010; Schuur and Haggard, 2011; Obhi, 2012).

The aim of the present EEG study was to explore the oscillatory brain dynamics underlying the selection and preparation of movements, and more specifically, to search for potential oscillatory activity differences between the externally and internally-driven mode of action selection. To limit the confounding factor of complexity between the two selection modes, simple unilateral left or right key presses were required as motor responses. The preparation phase was explored in a motor priming S1–S2 paradigm, which separated preparation from execution processes while matching timing factors across selection modes. A control task was additionally designed to evaluate the absence of specific motor preparation in the S1–S2 interval. We focused our analysis on the alpha (8–13 Hz) frequency band, known to be modulated not only by sensory stimuli and movements, but also by attention (Sauseng et al., 2005b; Thut et al., 2006), working memory (Jensen et al., 2002; Sauseng et al., 2005a), as well as internal tasks such as visual imagery (Cooper et al., 2003). In addition, we analyzed the lateralization index of alpha power in relation to internal and external motor preparation. In the context of willed action, the lateralization index of sensorimotor oscillatory activity constitutes a novel tool for exploring the dynamics of free selection and preparation of a motor response. Moreover, to examine further the neural mechanisms underlying the organization of action, we explored the relation between the preparatory pattern of alpha activity in each selection mode and the level of alpha lateralization in the Full condition, where movement preparation was achieved with the highest degree of certainty. Using this approach, we were able to depict distinct alpha cerebral patterns according to the level of motor readiness at the time of S2 presentation. These activation patterns were revealed to be similar in both internal and external modes of action selection.

## Materials and methods

### Participants

Thirty healthy volunteers (24.4 ± 2.5 years; 11 males) participated in the study. All participants had normal or corrected-to-normal visual acuity, were free of medication and none reported a history of major medical disorders, sustained head injury, psychiatric or neurological disorders, alcohol or drug abuse. They were all right-handed according to a twelve-item version of the Oldfield Edinburgh handedness inventory (Oldfield, 1971). They all provided written informed consent and the study was approved by the Ethics Committees of the Faculty of Psychology and Educational Science (University of Geneva) and of the University of Lausanne.

### Experimental design

**Figure 1:**
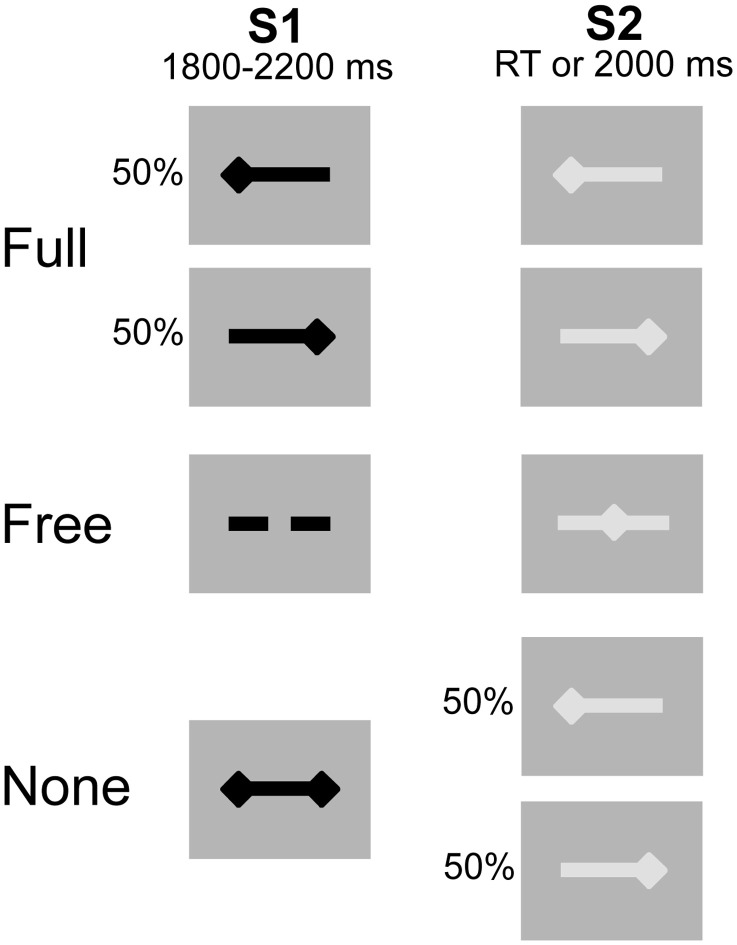
**Experimental conditions.** Preparatory signal (S1) in black, imperative signal (S2) in gray. After S2 offset, a blank screen was displayed for a duration varying between 1000 and 1500 ms. The 3 conditions were presented sequentially. Unilateral left- and right-sided diamonds for left and right side response, respectively. Double-sided diamonds (S1) for no information. Dashed bar (S1) and middle diamond (S2) for free selection and execution, respectively.

Participants were comfortably seated in front of a computer-controlled screen at a distance of 60 cm. The motor priming paradigm involved two types of visual stimuli, subtending a visual angle of 4.8° × 1°: a preparatory stimulus (S1) presented for an interval (foreperiod) varying between 1800 and 2200 ms, and an imperative stimulus (S2) lasting until the subject responded or 2000 ms elapsed. The inter-trial interval varied randomly between 1000 and 1500 ms (Figure 1). The tasks consisted of unilateral key presses with the left or right index finger under three different pre-cued conditions: (1) Full, where S1 provided complete advance information about response side; (2) Free, where S1 invited the subject to select the side of response and (3) None, where S1 was uninformative on response side. S2 provided complete response side information in the Full and None conditions and was neutral in the Free condition. Participants performed the Full, Free and None conditions successively (26 trials per condition) and repeated the experiment once (total of 52 trials per condition). The block design ensured that subjects remained concentrated within each condition, reducing errors and omissions. The intrinsic trial-by-trial variance of the choice-reaction-time paradigm helped to reduce the habituation and/or anticipation effects associated with fixed block designs.

### Data acquisition

Electroencephalogram (EEG) was continuously recorded during the experimental phase using 64 scalp electrodes at a sampling rate of 2048 Hz (BioSemiActiveTwo, Amsterdam, Netherlands). As reference, the system utilizes a feedback loop between two separate electrodes located over the posterior region. EEG channels were then referenced off-line to the mean voltage of all 64 channels (average reference). Electrode impedances were kept below 5 kΩ. The electro-oculogram was recorded using two pairs of bipolar electrodes in both vertical and horizontal directions. Visual stimuli and button presses were automatically documented with markers in the continuous EEG file.

### Data analysis

Reaction time was measured as the time interval between S2 onset and response key press. Accuracy was defined as the percentage of correct responses. Incorrect responses included errors (wrong key press) and omissions (absence of response within the 2000 ms-response window).

EEG data analysis was conducted using BrainVision Analyzer 2 software (Brain Products GmbH). Continuous EEG data were downsampled to 256 Hz and corrected for blinks and eye movement artifacts through an independent component analysis (ICA) (Jung et al., 2000). For EEG power analysis of the preparation interval, EEG data were segmented into S1-locked epochs of 5200 ms, starting 1000 ms before S1. Only the 1800 ms after S1 were analyzed (minimum foreperiod common to all trials). To obtain a precise evaluation of the signal preceding S2 for MRAA analysis, data were segmented into S2-locked epochs starting 2750 ms before S2. The epochs were automatically scanned for contamination by muscular or electrode artifacts (criteria for rejection: voltage step >50 μV/ms or peak-to-peak deflection within 300 ms intervals >200 μV) and the remaining trials were inspected visually to control for residual minor artifacts. Only artifact-free EEG trials corresponding to correct responses were analyzed, reaching the average number of 48 per subject in the Full and Free conditions and 47 per subject in the None condition.

A time-frequency (TF) analysis, based on a continuous wavelet transform of the signal (complex Morlet's wavelets), was performed between 8 and 13 Hz in 1 Hz steps (Tallon-Baudry et al., 1998). The mean power of the prestimulus interval was considered as a baseline level and subtracted from prestimulus and poststimulus power, independently for each frequency, providing a measure of relative power. For analysis of the preparation period, prestimulus interval corresponded to −650 to −150 ms before S1 onset. For MRAA analysis, prestimulus interval was selected between –2450 and –2100 ms before S2 onset (thus also preceding S1). TF analysis during the preparation period was performed in each condition for right and left hand responses combined. MRAA were obtained for each condition and response hand from relative power (P) at selected pairs of central electrodes (e.g., C3, C4) according to the following formula:

$${\text{MRAA}}_{\left(\text{C3},\text{C4}\right)}=(({\text{P}}_{\text{C4}}-{\text{P}}_{\text{C3}})\text{left hand}+({\text{P}}_{\text{C3}}-{\text{P}}_{\text{C4}})\text{right hand})/2.$$

Thus, the larger ERD over the region contralateral than ipsilateral to movement was reflected as a negative MRAA. Since the largest alpha-band MRAA was obtained for the CP3/CP4 pair, as previously reported (McFarland et al., 2000), further statistical analysis was restricted to this pair.

### Statistical analysis

RT and accuracy were compared across conditions using a one-way repeated measures ANOVA and *post-hoc* analysis used paired sample *t*-tests with a statistical threshold of *p* < 0.01.

EEG power was averaged in the alpha frequency range (8–13 Hz) within eight regions of interest (ROIs) in nine 200 ms time windows starting at S1 onset (0–1800 ms). Regions of interest were defined as follows: Left Motor (LM, C3, C5, CP3, CP5), Right Motor (RM, C4, C6, CP4, CP6), Left Premotor (LPM: FC3, FC5), Right Premotor (RPM: FC4, FC6), Left Parieto-occipital (LPO: PO7, O1), Right Parieto-occipital (RPO: PO8, O2), Midparietal (CUN: Pz, POz) and Midfrontal (SMA: Cz, FCz). Comparison of alpha relative power between conditions was performed using a 3-way repeated measures ANOVA, with Condition, ROI and Time as within-subject factors. *Post-hoc* analysis used paired sample *t-tests* with a statistical threshold of *p* < 0.01. For each condition and 200 ms time window, the CP3/CP4 MRAA difference from 0 was tested using one-sample *t*-tests with a statistical threshold of *p* < 0.01. To examine potential relationships between the preparatory alpha activity and the degree of sensorimotor alpha lateralization, the TF data were further analyzed using a tertile split. The MRAA amplitudes in the last 200 ms window preceding S2 in the Full condition were divided into low, medium and high values. Relative alpha power was then compared between the upper (high lateralizers, *N* = 10) and lower (low lateralizers, *N* = 10) tertiles, subjects falling into the medium tertile being discarded from analysis. In each Full, Free and None condition, a 3-way repeated measures ANOVA was performed with ROI and Time as within-subject factors and the Lateralization subgroup as a between-subject factor. *Post-hoc* analysis used independent sample *t*-tests with a statistical threshold of *p* < 0.01.

## Results

### Behavioral data

RT for correct responses were similar in the Full (320 ± 63 ms) and Free (318 ± 59 ms) conditions, and longer in the None condition (424 ± 63 ms). A significant effect of condition was evidenced on RT [*F*_(2, 58)_ = 132.71, *p* < 0.001]. *Post-hoc* analysis showed that RT in the None condition was significantly longer than in both Full and Free conditions (*p* < 0.001). In free-choice trials, subjects made a balanced proportion of right (51.1 ± 4.4%) and left (48.9 ± 4.4%) key presses. A high level of accuracy was observed, with percentages of correct responses of 99.8 ± 0.8% in Full, 99.6 ± 0.8% in Free and 98.6 ± 1.7% in None. Incorrect responses were exclusively omissions in Full and Free (no error by definition in Free) and included 93% of errors and 7% of omissions in None. There was a significant effect of condition on accuracy [*F*_(2, 58)_ = 8.30, *p* < 0.005] and *post-hoc* analysis showed that accuracy was significantly poorer in None as compared to each Full and Free condition (*p* < 0.01).

### Alpha power during the preparation period

**Figure 2:**
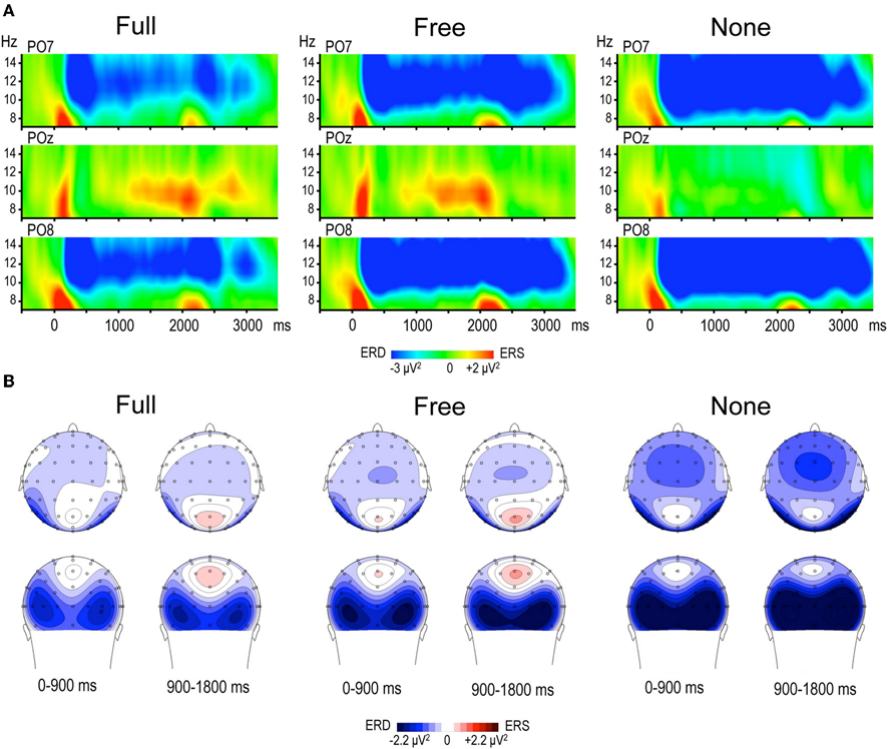
**(A)** Time frequency (TF) plots displaying the relative power of EEG signal between 7 and 15 Hz at posterior electrodes PO7, POz and PO8, for each motor preparation condition (left and right responses pooled). Time 0 denotes S1 onset, S2 onset occurring randomly between 1800 and 2200 ms. The preparation interval is accompanied by an alpha ERD at PO7 and PO8 in the three conditions, and by an alpha ERS at POz in Full and Free. **(B)** Topographic maps of relative alpha TF power (8–13 Hz) averaged across left and right responses during the preparation period, for each condition. The 1800 ms preparation period is subdivided into two 900 ms periods, each illustrated by two maps (top row: top scalp view; bottom row: back scalp view). Alpha ERD is globally larger in None, whereas midparietal alpha ERS is equally large in Full and Free.

**Figure 3:**
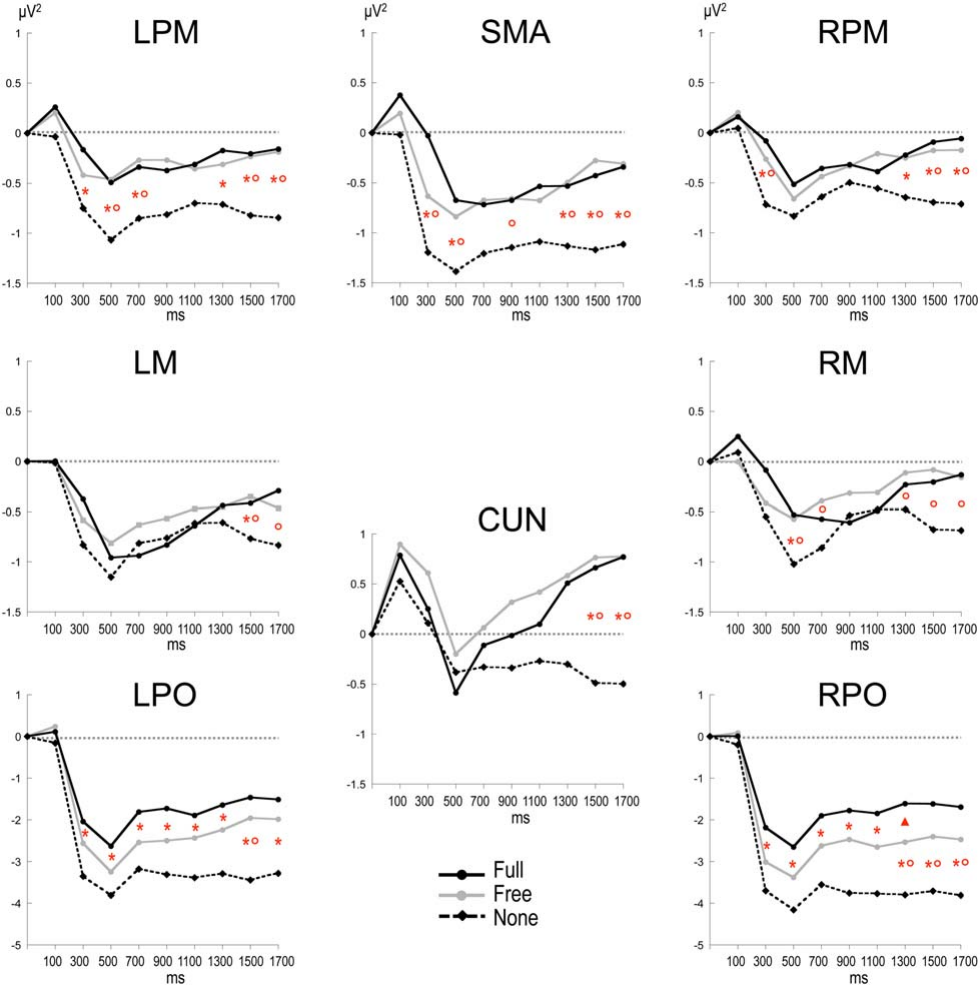
**Time plots of relative alpha power during the preparation period in the three conditions.** X-axis displays the center values of each 200 ms time intervals from 0 to 1800 ms after S1 (*x* = 0 corresponds to the baseline time window, −650 to −150 ms before S1). *Post-hoc* paired sample *t*-tests, *p* < 0.01: ^*^: None ≠ Full; °: None ≠ Free; ^*^°: None ≠ Full and Free; Δ: Free ≠ Full (*p* < 0.05). LPM: left premotor cortex; RPM: right premotor cortex; SMA: supplementary motor area; LM: left motor cortex; RM: right motor cortex; CUN: cuneus; LPO: left parieto-occipital cortex; RPO: right parieto-occipital cortex.

Alpha power within the preparation period differed substantially in intensity, topography and temporal course according to the nature of advance information, as expressed by a significant triple interaction between Condition, ROI and Time [*F*_(112, 3248)_ = 3.15, *p* < 0.001]. Two main observations were made (Figure 2). (1) A marked alpha ERD occurred bilaterally over the parieto-occipital regions during the whole foreperiod in all conditions. This alpha ERD was larger in the absence of advance information (None), of intermediate size when response had to be freely selected (Free) and smaller in the presence of advance instruction (Full). (2) An alpha ERS developed over the midline parietal region during the Full and Free foreperiods. Results of the *post-hoc* tests revealed that alpha ERD in Full and Free was significantly smaller than in None in all ROIs, generally at the end of the foreperiod (Figure 3). No significant ERD amplitude difference was observed between Full and Free conditions, although there was a tendency towards a larger alpha ERD in Free than in Full in the right parieto-occipital region between 1200 and 1400 ms (*p* < 0.05).

### Alpha MRAA

**Figure 4:**
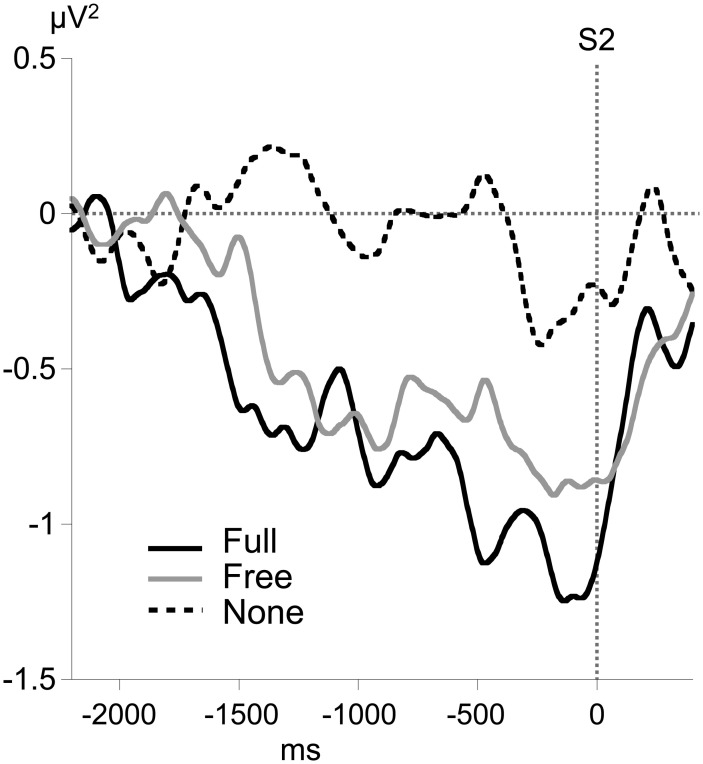
**Time course of motor-related amplitude asymmetry (MRAA) preceding S2 in the alpha band (8–13 Hz) for each condition, calculated from relative alpha power at CP3 and CP4 electrodes**.

No consistent alpha MRAA was obtained in the None condition (non-significant difference from 0). In contrast, alpha MRAA started at the beginning of the foreperiod in both Full and Free conditions and increased in amplitude until their peak at S2 onset, reflecting larger alpha ERD amplitude over the sensorimotor regions contralateral to movement (Figure 4). Full MRAA differed significantly from 0 by −1000 ms to S2 (*p* < 0.01), whereas Free MRAA differed significantly from 0 by −400 ms to S2 (*p* < 0.01). In the Full condition, a negative correlation was observed between the amplitude of midparietal alpha ERS and MRAA in the last 200 ms time window preceding S2 (Pearson coefficient: −0.47, *p* < 0.01). This correlation was not significant in the Free condition.

### Relation between preparatory alpha power and MRAA

**Figure 5:**
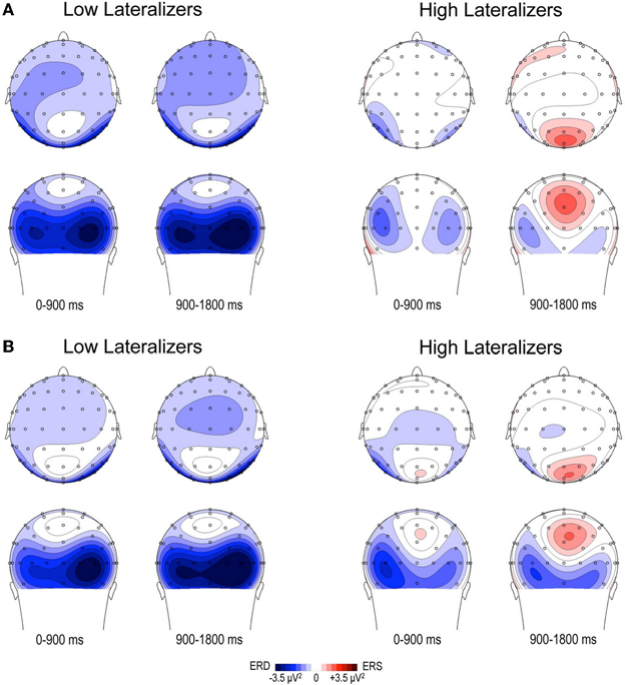
**Topographic maps of relative TF alpha power (8–13 Hz) averaged across low and high lateralizers during the preparation period.** The lateralization subgroups are obtained from the tertile split of MRAA values within the last 200 ms time window in the Full condition (see text). **(A)** Full condition, **(B)** Free condition. For each subgroup, two maps (top and back scalp views) are displayed per 900 ms time period. In both conditions, low lateralizers show predominantly right posterior alpha ERD, whereas high lateralizers display prominent midparietal alpha ERS.

**Figure 6:**
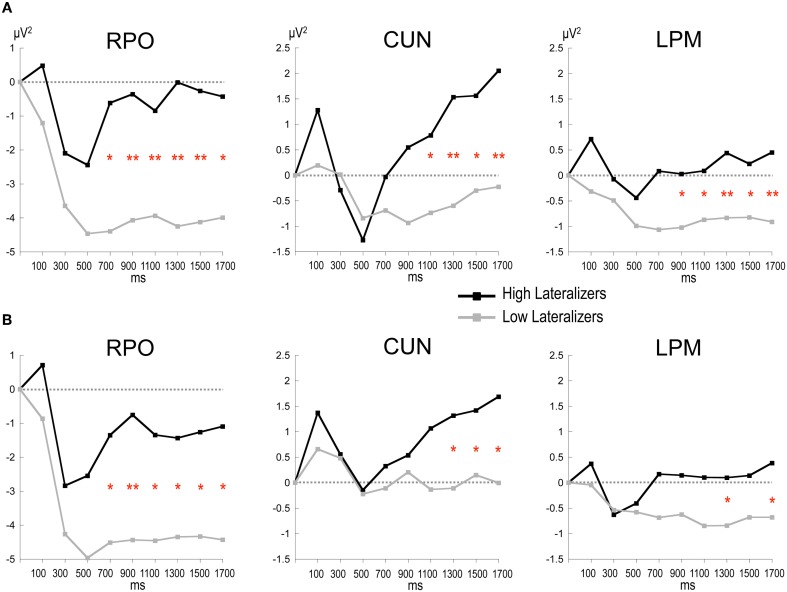
**Time plots of relative alpha power during the preparation period in subgroups of high and low lateralizers (see text for details). (A)** Full condition; **(B)** Free condition. Same x-axis convention and abbreviations as in Figure 3. *Post-hoc* independent sample *t*-tests: ^*^: *p* < 0.05; ^**^: *p* < 0.01.

In the Full condition, the two lateralization subgroups displayed distinct preparatory alpha patterns (Figure 5A), as confirmed by a significant Lateralization subgroup × ROI × Time interaction [*F*_(56, 1008)_ = 1.72, *p* < 0.001]. Compared with subjects with small MRAA amplitude (low lateralizers), subjects with large MRAA amplitude (high lateralizers) showed larger ERS over the midparietal region, in parallel with smaller ERD over the right parieto-occipital and left premotor regions (*post-hoc* independent sample *t*-tests, Figure 6).

In the Free condition, a similar distinction between alpha patterns was observed according to the degree of sensorimotor alpha lateralization (Figure 5B), confirmed by a significant Lateralization subgroup × ROI × Time interaction [*F*_(56, 1008)_ = 1.48, *p* < 0.01]. As in Full, high lateralizers displayed larger ERS in the midparietal region and smaller ERD in the right parieto-occipital and left premotor regions compared with low lateralizers. However, the differences between high and low lateralizers were not as large as in the Full condition (*p* < 0.05, Figure 6).

In the None condition, no significant difference in alpha power was observed between the lateralization subgroups.

## Discussion

The present study described the alpha oscillatory patterns in two conditions of advance movement preparation and one condition without specific motor preparation. In all these conditions, the preparatory period was associated with a widespread alpha ERD, dominating in the parieto-occipital region bilaterally. In agreement with the view that alpha ERD is an electrophysiological correlate of increased excitability in the cortical networks (Pfurtscheller and Andrew, 1999), the posterior alpha ERD observed during the S1–S2 preparation interval can be interpreted as reflecting visuo-attentional processes engaged in the preparatory set (Klimesch, 1999; Deiber et al., 2005; Thut et al., 2006; Klostermann et al., 2007). In our study, the presence of S1 visual stimulus throughout the whole foreperiod maintained visual activity, contributing to the sustained parieto-occipital alpha ERD. In the Full and Free conditions that permitted specific advance preparation, this alpha ERD was accompanied by an alpha ERS in the medial posterior region. Additionally, lateralized alpha activity was observed over the sensorimotor regions, as reflected by significant alpha MRAA, indicating contralateral engagement by the cortical motor areas in preparation for unilateral movement.

### Alpha activity in absence of specific motor preparation

Although no specific preparation could be achieved, alpha ERD amplitude was larger in the None than in both the Full and Free conditions. This suggests that instead of a passive process, a general activation state occurred in widespread regions to prepare for reaction at S2. The posterior maximum of this activation is concordant with regional cerebral blood flow data showing larger posterior parietal activation in conditions with absent or limited advance information as compared to full information about movement (Deiber et al., 1996). A difference of this kind was interpreted as a need for enhanced visuospatial attention to S2 for correct movement selection in conditions with restricted preparation. Few electro-cortical data are available on spectral power modulation during unspecified motor preparation, as most studies explore preparation intervals with variable degrees of response uncertainty, including the absence of response requirement. These studies have generally described larger alpha and beta ERD over sensorimotor regions in the S1–S2 interval when a response was required at S2 and when no uncertainty remained on the response (Kaiser et al., 2001; Alegre et al., 2004; Deiber et al., 2005; Klostermann et al., 2007; Tzagarakis et al., 2010; Sabate et al., 2012). However, no difference in the posterior regions but a reduced alpha ERD in the SMA was observed in the “None” condition compared with completely or partially pre-specified conditions for five possible spatiotemporal configurations of bimanual finger responses (Deiber et al., 2005). Hence, the alpha oscillatory pattern related to unspecific motor preparation may vary according to the characteristics of the upcoming motor response, and in particular to the mobilization of visuospatial attentional resources. Further investigation should determine whether large posterior alpha activation denoting high attentional level is more generally associated with a limited number of motor choices, and potentially high anticipatory behavior, as the present data tend to suggest.

### Alpha activity during externally and internally-driven motor preparation

The Full and Free conditions displayed comparable amplitude of alpha ERD in all cerebral regions, indicating that both modes of selection were activating the neuronal networks corresponding to motor preparation in similar ways. There was a tendency toward a larger alpha ERD in Free than in Full in the right parieto-occipital cortex, suggesting additional attentional resources associated with free choice. Moreover, in contrast with None, both Full and Free conditions were associated with a medio-posterior alpha ERS occurring in the second half of the foreperiod. During selective attention and working memory tasks, alpha ERS has been shown to develop over areas not engaged in the task, reflecting inhibition of task-irrelevant regions (Klimesch et al., 2007; Jensen and Mazaheri, 2010; Foxe and Snyder, 2011). A recent analysis of alpha oscillatory activity during working memory localized a midposterior alpha ERS in the cuneus (Brodmann areas 18/19), interpreted as the functional inhibition of visual areas no longer relevant to the task (Michels et al., 2008). In our paradigm, while S1 lasted for the whole delay, it was only in the two conditions where movement could be prepared that the midparietal alpha ERS developed. Moreover, its amplitude was proportional to the lateralization of sensorimotor alpha activity at the end of the foreperiod in the Full condition, suggesting that the inhibition of visual processing was linked to a state of motor readiness. In other words, once selection of movement is achieved, motor preparation processes would occur in parallel to inhibition of S1 visual guidance, which would no longer be necessary. This finding supports the view that a balance between engagement of motor areas and suppression of ongoing distracting visual information is taking place in the top-down control of motor preparation reflected in alpha-band oscillations (Jensen and Mazaheri, 2010; Foxe and Snyder, 2011).

The absence of alpha power difference between instructed and free motor preparation raised the question of the sensitivity of the method and/or the parameter under study. To our knowledge, alpha activity has not been formally compared between internal and external motor preparation states in delayed response tasks. In simple RT tasks performed by only six subjects, Tremblay et al. (2008) did not observe any clear-cut difference in alpha ERD preceding internal and external responses, although there was a wider choice of finger movements than in the present study and thus potentially more confounding factors linked to increased effort. Instead, they showed that the timing of beta activity was sensitive to the mode of action selection. In the same vein, ERP mapping showed identical electrical brain activity configurations within the S1–S2 interval for internal and external motor selection, the only differences being in their duration (Thut et al., 2000). Neuroimaging studies have also described a similar network of brain areas activated in externally- and internally-driven motor responses, with more activation for the latter in restricted cerebral regions such as the pre-SMA or anterior cingulate cortex (Deiber et al., 1996; Hoffstaedter et al., 2012), which cannot be easily resolved using EEG techniques. The present EEG data are consistent with the current literature in showing a global similarity of alpha activity in external and internal motor preparation, but fail to demonstrate a regional difference in alpha power between the two selection modes. Further studies should determine whether a distinction of this kind in selection control is reflected in other frequency bands and/or other parameters of oscillatory activity, such as phase synchronization (Schyns et al., 2011).

### Alpha motor-related amplitude asymmetry during motor preparation

The present data confirmed previous observations on the sensitivity of the alpha lateralization index to advance movement preparation. Early lateralization of alpha amplitude was observed over the motor regions following reliable advance information on response laterality, independently of response execution probability at S2 (de Jong et al., 2006; Gladwin et al., 2008). While replicating these findings, we also showed that the free condition displayed a significant alpha amplitude lateralization pattern, indicating that participants had indeed made an early selection of their movement when required to do so. Alpha lateralization reached statistical significance later in the Free than in the Full condition, suggesting that selecting movement side on internal decision took more time than it did on external guidance. This interpretation supports recent ERP results showing that the update of motor plans impacted differently on free and instructed choices and suggesting that, in free selection, multiple choices remained available until a late stage in motor preparation (Fleming et al., 2009). While RTs were the same in the two selection modes, as previously observed (Fleming et al., 2009; Hoffstaedter et al., 2012), our data showed that alpha lateralization reached a similar amplitude for internal and external motor selection at S2 occurrence, indicating comparable motor readiness. In addition, the significant alpha lateralization index in the Free condition where S1 was uninformative further supports the general link between alpha lateralization and motor processes rather than visuospatial attention shifts, which could potentially be triggered by S1 in the Full condition (Doyle et al., 2005). Lateralization of alpha ERD over motor cortical areas has also been repeatedly described during motor imagery, demonstrating the sensitivity of alpha oscillations to covert motor processes (Pfurtscheller and Neuper, 1997; McFarland et al., 2000; Nikulin et al., 2008). The present findings further emphasize the relevance of alpha MRAA in assessing the covert motor preparation processes involved in free choice.

### On the relation between global and motor-related alpha activity

In an attempt to explore further the relations between alpha oscillatory patterns, the degree of sensorimotor alpha lateralization and the mode of motor selection, we used the amplitude of the alpha lateralization index preceding S2 in the Full condition to split the subjects into high- and low- lateralizers. The Full condition served as reference, assuming that motor preparation would be achieved at the end of the foreperiod. The oscillatory alpha patterns were found to differ between the two subgroups, providing indications about underlying cerebral functioning. Furthermore, they replicated in the Full and Free conditions, indicating similarities in external and internal selection strategies. Two types of cerebral activation patterns could be distinguished. First, participants with large lateralization of sensorimotor alpha activity (high lateralizers) exhibited dominating midparietal alpha ERS, suggesting that they primarily relied on a strategy of motor activation/task-irrelevant visual activity suppression; they also showed less activation in the premotor regions, probably subsequent to activation of the motor regions. Second, participants with weak lateralization of sensorimotor alpha activity (low lateralizers) displayed predominant right parieto-occipital alpha ERD, indicating the primary recruitment of right dominant visuospatial attentional resources (Mesulam, 1981). These observations were consistent with our initial finding that, for a simple between-hand choice, no fundamental difference could be observed in alpha activity pattern when the selection was internal as opposed to external. Instead, they revealed that, independently of the selection mode, distinct cerebral alpha patterns can be observed in relation to the lateralization of sensorimotor alpha activity preceding the response. We distinguished a *motor alpha pattern*, characterized by elevated lateralization of sensorimotor activity and concomitant suppression of visual activity, from an *attentional alpha pattern* featuring reduced sensorimotor lateralization paralleled by increased activation of posterior attentional networks. In the young population presently studied, both alpha patterns led to similar RTs in each external and internal selection mode, indicating that they were equally effective on motor execution. Alternatively, the two alpha patterns are likely to reflect distinct preparatory cerebral strategies, the functional relevance of which remains to be clarified.

## Concluding remarks

It is of particular interest that we were able to distinguish different alpha patterns during motor preparation in relation to the amplitude of alpha lateralization before movement, but not in relation to the mode of action selection. This observation also indicates that motor-related alpha oscillations are primarily linked to production processes, rather than to higher executive control of motor functions. The majority of electrophysiological (Okano and Tanji, 1987; Romo and Schultz, 1987; Mushiake et al., 1991) and neuroimaging (Deiber et al., 1991, 1996, 1999; Jahanshahi et al., 1995; Cunnington et al., 2002) evidence has converged to date in distinguishing a medial frontal network primarily involved in self-generated action from a dorsal premotor network implicated in externally-triggered actions. However, the observation that the anatomical distinction was not always absolute, as well as the greater complexity associated with self-generated actions, has recently opened a debate on the relevance of the distinction between self-generated and externally triggered actions (Nachev et al., 2008; Passingham et al., 2010; Schuur and Haggard, 2011; Obhi, 2012). The present results, although they need to be complemented by source localization procedures and extended to various motor outputs, do at least suggest that this kind of distinction cannot be demonstrated when examining the surface reactivity of the alpha frequency band in preparation for simple unilateral finger responses.

### Conflict of interest statement

The authors declare that the research was conducted in the absence of any commercial or financial relationships that could be construed as a potential conflict of interest.
